# Virtual World Café Method for Identifying Mental Health Research Priorities: Methodological Case Study

**DOI:** 10.3390/ijerph19010291

**Published:** 2021-12-28

**Authors:** Michelle Banfield, Amelia Gulliver, Alyssa R. Morse

**Affiliations:** Centre for Mental Health Research, Research School of Population Health, The Australian National University, Acton, ACT 2601, Australia; amelia.gulliver@anu.edu.au (A.G.); alyssa.morse@anu.edu.au (A.R.M.)

**Keywords:** mental health, internet, methods, qualitative research

## Abstract

People with lived experience of mental health problems as both consumers and carers can bring significant expertise to the research process. However, the methods used to gather this information and their subsequent results can vary markedly. This paper describes the methods for two virtual World Cafés held to gather data on consumer and carer priorities for mental health research. Several methodological processes and challenges arose during data collection, including the achieved recruitment for each group (*n* = 4, *n* = 7) falling significantly short of the target number of 20 participants per group. This led to departures from planned methods (i.e., the use of a single ‘room’, rather than multiple breakout rooms). Despite this, the participants in the virtual World Cafés were able to generate over 200 ideas for research priorities, but not identify agreed-upon priorities. Virtual World Cafés can quickly generate a significant volume of data; however, they may not be as effective at generating consensus.

## 1. Introduction

There is increasing recognition of the expertise that people with lived experience of mental health problems as both consumers and carers can bring to the research process [1,2,3]. The value of this expertise now provides a platform for consumers and carers to move past tokenism and consultation, towards co-design and meaningful partnerships in research [4]. The inclusion of consumer and carer views at the earliest stages of research design is critical to ensuring that their perspectives are at the cutting-edge of research [5]. In addition, it is important to ensure that we meet the ongoing objective of regularly updating their recorded views on priorities for mental health research, which enables the field to be responsive to the needs of the people for which it aims to serve [5].

The COVID-19 pandemic has seen dramatic global increase in the use of technology to facilitate many processes that usually involve face-to-face communication. This includes a range of activities, such as teaching [6], and the provision of medical treatment, ranging from services for neurology [7] and psychology [8,9] to midwifery and occupational therapy [10], physiotherapy [11], and yoga classes [12]. Research has also been forced to move online so as not to lose momentum, but evidence is emerging that this presents new challenges, as switching to remote data collection is not always seamless and can be difficult for participants [13,14].

The continuation of research that is normally facilitated face-to-face, such as focus groups [15] and nominal group methods [16,17], has also been adapted with technology. Researchers have considered that there are advantages of online video-based discussion groups, which include participants who potentially feel more comfortable to express their opinion, preventing group speak, and the ability to access those who would not normally attend a group in person [15]. Some disadvantages include being less able to detect body language (e.g., if a participant expresses an emotional response to a question, which has implications for safety), technical issues in using the technology, and limits on those who have access to technology [15].

Developed recently, the World Café method is a powerful way of facilitating group discussions [18]. It is particularly useful for gathering the views of large groups to creatively work together in a single conversation [19], where you already have all the experts in the “room”. World Cafés typically involve bringing together small groups of people at tables in a comfortable environment, much like a café, to discuss a particular issue [20]. After a set period of time, participants are shuffled to new tables with new issues and new participants, and then the process is repeated several times [20]. Thus, the World Café method can be relatively easily adapted for use online. A recent study, conducted by McKimm et al. [21] during the COVID-19 pandemic, demonstrated the feasibility of using this method for health educators from across the world to discuss educational initiatives to promote online learning [21]. The researchers reported several issues with the method that related primarily to technological difficulties, audience size, and different time zones [21]. However, they also noted many benefits to the process as it was able to gather the views of geographically dispersed experts to find solutions, and provided a safe space to share challenges and opportunities [21]. There is much that can be learned from established research techniques using new technologies; however, there remains little research describing the implementation, challenges, and benefits of the adaptation of established methods. Thus, the purpose of the current paper is to describe the methods for virtual World Cafés held to gather data on consumer and carer priorities for mental health research. The World Cafés seeking mental health research priorities were conducted as part of a larger, multi-method study that aimed to develop a national research agenda for disability research in Australia (Smith-Merry et al., forthcoming). The current paper focuses on methodological processes and challenges; study findings will be presented elsewhere.

### Rationale

The current study was designed to update and build on previous priority-setting undertaken by our group [5]. The original priority-setting research consisted of a half-day face-to-face forum conducted in 2013 in the Australian Capital Territory (ACT), combining small group discussions to generate and prioritize research topics, and large group discussion of inclusive methods [5]. Our 2017 update comprised a national online survey in Australia, where participants could rate and rank the topics developed in the 2013 study [5]. For the current study, we sought to repeat the original forum process to renew consumer and carer priority-setting for Australian mental health research. Simultaneously, our work informed two further national priority-setting processes: a national disability research agenda for the National Disability Research Partnership; and a roadmap for translational mental health research being developed by The ALIVE National Centre for Mental Health Research Translation, a new National Health and Medical Research Council special initiative to progress collaborative mental health research at scale in Australia. In the COVID-19 context, instead of face-to-face meetings, we used an online format, comprising Zoom video conferencing (Zoom Video Communications Inc., San Jose, CA, USA), and an online polling platform, Slido (sli.do s.r.o, Bratislava, Slovakia). The following sections describe how we planned to execute the study, the challenges we encountered, and the ways in which we were able to respond to these challenges to provide a good experience for participants whilst remaining within the bounds of our ethical approval.

## 2. Materials and Methods

### 2.1. Recruitment and Consent Process

Participants were recruited through an advertisement distributed to our networks of consumer and national carer organizations (e.g., ACT Mental Health Consumer Network, Carers Australia) on our research group webpages and through social media.

Potential participants contacted the researchers to register their interest and were provided with the information sheet and consent form. To manage active consent and access to the Zoom World Cafés whilst maintaining confidentiality, we created a password-protected consent register, stored on university servers accessible only to the named researchers. When participants returned a scanned or photographed copy of the signed consent form, they were added to the consent register and provided with the private Zoom meeting link, which they were asked not to share with others. Recognizing that some people may have wished to use a pseudonym in the sessions, the consent form also asked participants for the name they intended to use on Zoom to facilitate the matching of consent. The waiting room feature of Zoom was enabled and people were only admitted to the World Café after researchers had checked the consent register. If a name without recorded consent appeared in the waiting room to join the World Café, a member of the research team privately messaged the person to check if we had recorded consent under another name, which happened with a small number of people at each session. We also had information sheets and consent forms to conduct on-the-spot consent via email for anyone whom we did not yet have recorded consent, but this was not needed.

We aimed for a total of 40 participants across two World Cafés (one held during business hours, and one in the evening), but recruitment fell short of what was planned. A total of 15 people consented to take part, of whom 11 people participated across the two World Cafés: 4 in the first and 7 in the second. Due to project time constraints and the volume of information generated in these two discussions, we chose not to conduct any further recruitment. No specific demographic data were collected; however, during the course of discussions, we established that people’s age and gender significantly varied, and that they were from the eastern Australian states, including New South Wales, Victoria, Queensland, and the ACT.

### 2.2. Virtual Discussions

The World Cafés were scheduled for 2.5 h, including breaks, and were held in April 2021. Prior to commencing the discussions, the facilitators ran through the housekeeping script to familiarize participants with key Zoom features, remind them about voluntary participation and confidentiality, agree on principles for the nature and content of discussions, and describe how to seek support if needed. A registered psychologist was available to privately support and refer any participants who became distressed, using the breakout room feature. Whilst it was not expected that discussing mental health research priorities would cause distress, this mechanism was put in place as a safeguard against unintentional triggering, for example by recalling a distressing event or hearing another’s story as a background to where mental health research should focus. This support was not accessed by any of the participants.

We planned four sessions: three rounds of small group discussions using the Zoom breakout room feature to create discussion “tables”, and one final large group discussion.

Discussions were framed by the following questions:

1. **What are the main issues you see as important in mental health in Australia?**

*Prompts:* What are the issues or problems that are important to you or the people you support? Are there any potential ways that these issues could be improved for you, or for the people you support?

2. **What sort of research would you like to see prioritized in a national research agenda?**

*Prompts:* What things do the government or other agencies need to know more about so they can better address your and your family’s needs? Is there a specific program, service, or treatment that you think should be evaluated? Is there a particular illness or group that we should focus on?

3. **How do you currently engage with research?**

*Prompts:* What features of research do you think make it useful for you or for others? How do you find out about participating in mental health research? How would you like to be informed about how to help with being involved in conducting research? How would you like to engage with research in the future?

The lead researcher (author MB) hosted the Zoom sessions and had control over assigning participants to rooms, with one researcher assigned to each breakout room as facilitator, assisted by an observer/note taker. We planned for participants to be assigned to rooms and moved between breakout rooms by the host to ensure that everyone had the opportunity to contribute to each question and to interact with different participants. Each session was scheduled to last for 20 min, with 5 min in between to facilitate moving participants and allow comfort breaks. When participants commenced in a new breakout room, they were to be informed of the discussion in that room so far and asked for further comments and additions to the question. This was designed to allow both reinforcement of key issues already raised and the opportunity to add novel areas.

As recruitment fell short of the target, the procedure was modified. Instead of creating small group “tables” using breakout rooms, all participants remained in the main discussion room for all questions. This changed the nature of the method to be more like a virtual nominal group [16] than a World Café, as the interchange of discussion groups was removed. However, as described in the next section, this did not affect the overall success of the priority generation.

At the conclusion of the small group sessions, a 20-min break was planned to allow participants and researchers to regroup in preparation for the final large group session. As described below, the purpose of this final session was to clarify and confirm the topics raised against the discussion questions before voting on priority areas.

### 2.3. Polling Platform

In our previous work [22], participants have emphasized the importance of privacy and confidentiality when discussing their experiences with mental health issues. The purpose of this study was to direct discussions to agreed written topics rather than analyze discussions in depth, so we decided to increase participant comfort regarding confidentiality by not recording sessions. Instead, to facilitate accurate capture of people’s ideas and engage them with the tasks, we used a web-based and interactive Q&A as well as a polling app, called Slido, (sli.do s.r.o, Bratislava, Slovakia) that encourages participation in virtual events. The app protected the identity of participants, as it requires no downloads or disclosure of personal information to interact with the tools. Participants followed a link provided within the Zoom chat and entered the unique event ID to access the interactive tools for the discussion session. Participants entered words and phrases in response to the research questions, which then automatically created a “word cloud” in Slido. Participants then interacted with the word cloud in real time by re-entering words or phrases already present to increase their emphasis (up-voting) or enter further words to expand the cloud. Participants reported that they felt more comfortable raising issues and up-voting other suggestions because, unlike a normal discussion group, their contributions were anonymous unless they chose to reveal their additions.

Facilitators encouraged discussion about research topics emerging in response to the emphasis suggested by the cloud at several points in each session. This helped to clarify topics and allowed note takers to capture further contextual information for later analysis. It also helped to address a limitation of the Slido word cloud tool, which was only able to display a finite number of words and phrases entered at any one time. Longer entries had the potential to make other topics disappear off participants’ screens, although they remained visible to the researchers in the control panel and were captured in the final topic lists.

Discussing the emerging topics at regular intervals drew participants’ attention to the range of ideas being entered, but there were striking differences between the amount of discussions and the number of topics each group generated. The first group generated 46 topics, and the second group generated 154 topics. There was a large amount of verbal discussion in the smaller group (*n* = 4), whereas in the larger group (*n* = 7), there were many more topics generated and much of the verbal discussion focused on the topics being entered into Slido. Participants commented that this flexibility helped them contribute to ideas generation without feeling the usual pressure to be an active part of group discussions. Note takers ensured that topics developed during verbal discussions were entered into the Slido tool.

At the conclusion of the discussions on the three questions, the researchers downloaded the data from each of the word clouds into Microsoft Excel, and then copy and pasted the full list into two polls to identify the most and least important topics. Initially, two researchers attempted to copy and paste topics simultaneously, but doing this in real time unfortunately resulted in data being overwritten in the Slido control panel. Thus, the data had to be downloaded into Excel from Slido by one researcher, and then copied and pasted back into the Slido polls for participants to rate. There was some trial and error involved to successfully paste the lists back into Slido, as it initially appeared that topics had to be pasted individually. This was extremely labor-intensive for the 45+ generated in the first group, and the 150+ in the second. However, careful selection and copying of a set of cells from Excel allowed pasting of the entire list back to Slido, automatically populating a set of poll options. When this had successfully been completed, the polls were made live and participants were asked to rate the one topic they thought was the *most important*, and the one topic they thought was the *least important*. Poll options were set to allow only one answer to each, forcing participants to consider their highest and lowest priority.

Participants in each group all identified unique priorities, with no single priority being voted as most or least important by more than one participant in both groups.

## 3. Results and Discussion

Despite some challenges and departures from planned methods, our virtual World Cafés successfully generated 200 mental health research priorities and, therefore, achieved the project’s main aim. Key departures from our planned research included the single online room, and the smaller groups, which made the adapted approach closer to a nominal group method [16,17]. However, the lack of agreement on the top priorities means the activities also fell somewhat short of being called a consensus method. Each of these key points are discussed in more detail below.

Despite the modified World Café method and the small groups of participants, the number of priorities exceeded expectations based on our previous priority-setting work [5]. The ideas generation phase of this previous work was a face-to-face forum of 25 participants, who generated more than 80 research topics. Similar research on priority-setting for mental health research in Chile using multiple methods of interviews, focus groups, and a web survey (total *n* = 54) identified 155 research topics [23]. It is possible that the online methods enabled people in our current study to be more open with their ideas. The simplicity and engaging interactivity of the Slido tools also may have encouraged participants to brainstorm ideas rather than engage in in-depth discussions. This type of ‘silent idea generation’ is usually conducted as part the nominal group method rather than World Café, and seems to generate a range of ideas well in the online format [16]. This may be a powerful tool to enable people with lived experience to contribute to early research design. This idea is further supported by the notable differences between the two groups and their balance of verbal discussion and focus on producing topics. The group with the larger number of participants generated three times as many ideas with less than twice the number of participants. Whilst this may have been due to differences in individual and group dynamics between the two Cafés, it is also possible that the larger size of the second group encouraged participants to enter their thoughts anonymously rather than discussing them openly. The size of the larger group (*n* = 7) approached the upper recommended limit for focus groups, where up to eight participants are usually recommended so that each participant’s opportunity to share is not inhibited by large group dynamics [24].

This is an important consideration in using polling or whiteboard tools as adjuncts to discussions. The lack of sufficient participants to create multiple Café ‘rooms’ [20] is likely to have impacted our ability to create a rich verbal discussion and to have multiple participant views on each single topic. However, our main purpose was to allow people to generate ideas rather than engage in lengthy discussions. This meant that the relative lack of discussion and the possibility that the tools became the focus of the sessions were not problematic. Studies using methods similar to World Café to generate discussion and consensus may need to consider limiting the availability of online polling tools to discrete sessions in order to facilitate these purposes.

Our major methodological departure from what was planned was to remove the usual World Café small group discussions similar to the McKimm et al. [21] study, and instead keep all participants in the one virtual room for all sessions. It is possible that we could have delayed the Cafés and extended recruitment to generate sufficient numbers of participants to proceed with multiple rooms. However, deadlines for contribution to an external project and the need to respect the commitment of early volunteers to the advertised times made this unappealing. Our approach proved sufficiently flexible to successfully change in response to needs and limitations in real time, which we consider a strength.

Our final methods of discussing, collecting, and refining topics more closely resembled an online nominal group method [17], but there was no consensus on the topics nominated as the most and least important. While consensus was not really expected based on our previous studies [1,5,25], the dispersed effect was accentuated by the high number of available ideas to rate enabled by the software, combined with the low number of participants. Our previous studies involved the use of voting, as well as Likert-type rating scales and ranking [1,5,25], to try to achieve priorities, all of which proved challenging for participants who did not want to rate or rank one area as “more important” than another. For the current study, we tried to simplify the task by asking for just two choices: the most and least important. However, forcing participants into choosing just one of the 45+ (group 1) or 150+ (group 2) possibilities meant that a maximum of 11 ideas could be endorsed as most important, and participants commented that this was too difficult and too much responsibility. Consistent with the comments in our earlier work [5], participants noted that the Australian mental health system is poor, and that everything they had discussed and listed during the session was critical to mental health reform and research. They did not want us to focus solely on the ones they had nominated in the forced-choice poll.

To inform the national disability research agenda, we undertook a thematic analysis following the completion of both virtual World Cafés. This process served to make the findings more manageable for reporting in the context of broader research priority-setting. We also compared the specific topics developed in the current study with those developed in our previous work to explore how specific issues may have evolved over time, and contribute to the roadmap project for the ALIVE National Centre for Mental Health Research Translation. The results of these analyses are reported elsewhere (Banfield et al., forthcoming).

A limitation of the current study is that we did not identify people’s socioeconomic or cultural backgrounds to establish our reach. As a small-scale consultation exercise, we did not aim for representation in this study. Broader reach and further opportunities for underrepresented groups to generate ideas are the objectives of the larger priority-setting work within which this study sits, as described above. However, this means that specific considerations for conducting virtual World Cafés with socially disadvantaged or culturally diverse groups are unknown.

Recent work in low- and middle-income countries suggests that some groups may experience structural and cultural barriers to technology-based research, even when it is necessary [13,14]. Thus, it is important to explore means of inclusive research that enable diverse voices to be heard. This is especially the case in mental health consumer and carer priority-setting where the aim is to give voice to those with knowledge from their experiences of the system, but who are traditionally silenced. Research that specifically targets people from underrepresented groups, such as Aboriginal and Torres Strait Islander peoples, and uses culturally appropriate methods is still required, and may be better suited to face-to-face methods than online. In the new environment created by COVID-19, which has forced a reliance on technology, we need to actively seek out the groups who may be marginalized by this shift to develop further novel ways of enabling their participation.

## 4. Conclusions

Virtual World Cafés or discussion groups also use online polls, such as the ones described in this study, and can generate large amounts of data in short periods, but they may not be suitable to generate a consensus or priority list. They are a powerful way of enabling participants to contribute in a safe and anonymous manner, particularly those who may otherwise struggle with contributing to group discussions. This makes them a useful addition to the toolbox for the active involvement of people with lived experience of mental health problems in the research process. However, they may be better treated as idea-generating activities that explore participants’ breadth and depth of knowledge, rather than trying to craft a smaller number of agreed areas. A thematic analysis to organize specific topics into broad thematic areas is useful, but from the perspective of the scope of mental health research, there is no absolute need to have a small number of ranked topics to guide the process. A “menu” of possibilities that consumers and carers say are all important is an excellent agenda against which to work.
